# Identification of the Transmembrane Glucose Regulated Protein 78 as a Biomarker for the Brain Cancer Glioblastoma Multiforme by Gene Expression and Proteomic Studies

**DOI:** 10.4172/2155-9589.1000126

**Published:** 2014-02-15

**Authors:** HN Banerjee, G Hyman, S Evans, V Manglik, E Gwebu, A Banerjee, D Vaughan, J Medley, C Krauss, J Wilkins, V Smith, A Banerji, J Rousch

**Affiliations:** 1 Department of Natural Science, Elizabeth City State University, Elizabeth City, NC, USA; 2 Department of Mathematics and Computer Science, Elizabeth City State University, Elizabeth City, NC, USA; 3 Department of Pharmaceutical Sciences, Elizabeth City State University, Elizabeth City, NC, USA

**Keywords:** Glioblastoma multiforme, Astrocytoma, 2D-DIGE, Mass spectrometry, Proteomics, Microarray

## Abstract

The prognosis of patients with Glioblastoma Multiforme (GBM), the most malignant adult glial brain tumor, remains poor in spite of advances in treatment procedures, including surgical resection, irradiation and chemotherapy. Genetic heterogeneity of GBM warrants extensive studies to gain a thorough understanding of the biology of this tumor. While there have been several studies of global transcript profiling of glioma with the identification of gene signatures for diagnosis and disease management, translation into clinics is yet to happen. In the present study, we report a novel proteomic approach by using two-dimensional difference gel electrophoresis (2D-DIGE) followed by spot picking and analysis of proteins/peptides by Mass Spectrometry. We report Glucose Regulated Protein 78 (GRP78) as a differentially expressed protein in the GBM cell line compared to human normal Astrocyte cells.

In addition to proteomic studies, we performed microarray analysis which further confirmed up regulation of GRP78 in GBM cells compared to human normal Astrocyte cells.

GRP78 has long been recognized as a molecular chaperone in the endoplasmic reticulum (ER) and can be induced by the ER stress response. Besides its location in the ER, GRP78 has been found in cell plasma membrane, cytoplasm, mitochondria, nucleus and other cellular secretions. GRP78 is implicated in tumor cell proliferation, apoptosis resistance, immune escape, metastasis and angiogenesis, and its elevated expression usually correlates with a variety of tumor micro environmental stresses, including hypoxia, glucose deprivation, lactic acidosis and inflammatory response. GRP78 protein acts as a centrally located sensor of stress, which senses and facilitates the adaptation to the tumor microenvironment.

Our findings showed differential expression of this gene in brain cancer GBM and thus confirm similarities in findings in existing transcriptional and translational studies. Thus, these findings could be of further importance for diagnostic, therapeutic and prognostic approaches for dealing with this highly malignant cancer.

## Introduction

Glioblastoma multiforme (GBM) is the most common and most aggressive of the primary brain tumors with pathologic hallmarks of necrosis and vascular proliferation. The current World Health Organization classification of primary brain tumors lists GBM as a grade IV (malignant) astrocytoma [1]. Astrocytoma is one of the three distinct types of gliomas in the brain, although mixed cell types occur as well. GBMs are highly malignant, infiltrate the brain extensively and at times may become enormous before turning symptomatic. Among primary brain tumors, malignant astrocytomas are the most common in all age groups (however, among all brain tumors, metastases are the most common). GBMs are the most common primary brain tumors in adults, accounting for 12-15% of intracranial tumors and 50-60% of primary brain tumors.

Morbidity is a function of tumor location, progression, and pressure effects. The overall prognosis for GBM has changed little in the past two decades despite major improvements in neuroimaging, neurosurgery, radiation treatment techniques and supportive care. Few patients with GBM survive longer than three years and only a handful survive five years. Previously reported long-term survivors of GBM may be patients diagnosed with GBM who actually harbor low-grade glioma, pleomorphic xantho astrocytoma, ganglioglioma, or other lesions. Occasional patients with a single necrotic, demyelinating plaque of multiple sclerosis also may be misdiagnosed with GBM, especially if only CT scans are obtained.

The diagnosis of GBM is currently based on histological examination of brain tumor tissues after radiological characterization and surgical biopsy. These approaches are successful in classifying and grading tumors in most cases, but in many situations these techniques do not allow accurate prediction of prognoses and therapeutic responses. The situation may be further complicated by the small size of some diagnostic biopsy samples. There is, therefore, a critical need to improve the diagnosis of these brain tumors to both improve current therapeutic management strategies and form a basis for the evaluation of novel approaches.

The ability to characterize tumors comprehensively at the molecular level raises the possibility that diagnosis could be based on molecular profiling with or without histological examination, rather than solely on histological phenotype. The development of novel genomic and proteomic techniques will help in identification of such diagnostic and prognostic molecular markers.

Since proteomes directly regulate disease phenotypes [2-4], proteomic study is an effective approach in determining proteomic aberrations that must exist in conjunction with any type of disease. Two-dimensional difference gel electrophoresis (2D-DIGE), introduced in 1997 [5], is a high performance and accurate proteomic technology. In this this technique, a mixed-sample internal standard is used to determine and quantify human proteins which reduces inter-gel variability and simplifies gel analysis. Although 2D-DIGE is based on two-dimensional gel electrophoresis, specifically employing a multiplex detection system, the technique solves many drawbacks of classical 2D-PAGE [6-10]. The 2D-DIGE technique allows quantitative protein expression profiles across many clinical specimens to be obtained in a reproducible and high-throughput manner and with greater detection sensitivity of low abundant proteins. Additionally, 2D-DIGE in conjunction with high sensitive fluorescent dyes enables proteomic study on laser micro-dissected tissues, thereby further increasing the accuracy of proteomics observations [11-13].

Mass spectrometry and the use of gene and literature databases as a follow-up procedure to 2D-DIGE, allow further characterization of proteins that are apparently up-regulated in GBM cells. Bioinformatics approaches can determine the proteomic signatures responsible for the important clinico-pathological features and identify a small number of key proteins, which will be candidates for disease markers and therapeutic targets [14-16]. Combination of 2D-DIGE, mass spectrometry and bioinformatics approach will continue to develop into more powerful tools for disease proteomics. The efforts to understand the overall feature of proteome by bioinformatics approach to 2D-DIGE data, together with the integrated information of the individual proteins identified by 2D-DIGE, will give us novel molecular backgrounds of the diseases [17,18].

Molecular diagnostics is a rapidly advancing field in which insights into disease mechanisms are being elucidated by use of new gene-based biomarkers. Until recently, diagnostic and prognostic assessment of diseased tissues and tumors relied heavily on indirect indicators that permitted only general classifications into broad histologic or morphologic subtypes and did not take into account the alterations in individual gene expression. Global expression analysis using microarrays now allows for simultaneous interrogation of the expression of thousands of genes in a high-throughput fashion and offers unprecedented opportunities to obtain molecular signatures of the state of activity of diseased cells and patient samples. Microarray analysis may provide invaluable information on disease pathology, progression, resistance to treatment and response to cellular microenvironments and ultimately may lead to improved early diagnosis and innovative therapeutic approaches for cancer.

In this study, we took a novel approach of identifying differentially expressed proteins in GBM cells compared to human normal Astrocyte cells by using 2D-DIGE coupled with mass spectrometry (proteomic approach) and also using microarray technique to analyze the transcriptome specifically differentially expressed genes between cell lines.

## Materials and Methods

### Cell culture

Human normal astrocytes cells were a kind gift from Dr. K. Pahan of University of Nebraska Dental School (NE, USA), HTB15 human Astrocytoma cells were purchased from American Type Culture Collection (VA, USA). Astrocyte cells were cultured in DMEM-F12 medium supplemented with 10% calf serum and antibiotics penicillin-streptomycin (20μl/L of medium), 37°C in a carbon dioxide incubator. Astrocytoma cells were cultured in Leivowitz-15 medium (L-15 medium) supplemented with 10% calf serum and antibiotics penicillin-streptomycin under conditions similar to normal Astrocyte cultures.

### Two-dimensional difference gel electrophoresis (2D-DIGE)

Two-dimensional DIGE was performed at Applied Biomics (Hayward, CA, USA) following typical methods [19,20]. Briefly, cell lysates, were denatured by equal volume addition of lysis buffer containing 7M urea, 2M thio urea, 4% 3-((3-cholamidopropyl)dimethyl ammonio)-1-propanesulfonate(CHAPS) followed by addition of 30 mM Tris-HCl, pH 8.8, at a 5:1 volume ratio lysis buffer: plasma. Lysate samples were normalized using total protein as determined by Lowry protein estimation method. Next, samples were labeled with CyDye DIGE fluors developed for fluorescence 2D-DIGE technology (Cy3 and Cy5, GE Healthcare, CT, USA) and incubated in dark on ice, 30 min. The labeled samples were then subjected to isoelectric focusing (IEF) on a 13-cm precast non-linear immobilized pH gradient strip (pH 3-10, Amersham Biosciences, Buckinghamshire, UK) using an Amersham Pharmacia IPGPHOR unit with a power supply (EPS3501XL) in gradient mode. Next, the samples were separated by sodium dodecyl sulfate polyacrylamide gel electrophoresis (SDSPAGE) in the second dimension based on size. The gels were scanned using Typhoon Trioscanner (Amersham Biosciences) and fluorescent dye signals corresponding to individual samples converted to black and white images for analysis using Image Quant and DeCyder software (Amersham Biosciences).

### Mass spectroscopy (MS)

Based on 2D-DIGE assessment, proteins showing statistically significant differences in intensity between Astrocytes and Astrocytoma cells were excised from the gel using an Ettanspot picker (Amersham Biosciences), digested with trypsin (Promega Corporation, WI, USA) at 37°C, extracted with 2% trifluoro acetic acid and 40 μl of acetonitrile, and desalted with a C-18 ZipTip (Millipore Corp., MA, USA). Each sample was mixed with matrix buffer and spotted onto a MALDI plate. MALDI-TOF MS was performed using the ABI4700/ABI4800 (Applied Biosystems, CA, USA) proteomic analyzer according to manufacturer’s instructions. The top ten most abundant peptides noted during 2D-DIGE were further analyzed using MS/MS (two variable modifications, carbamido methyl and oxidation, one missed cleavage; precursor tolerance,100 ppm; MS/MS tolerance, 0.3D, peaks in MS and MS/MS spectra were analyzed for similarities using GPS explorer equipped with MASCOT search engine and NCBI and Swiss Prot protein databases.

### Microarray analysis and gene expression profiling

#### RNA sample preparation

Total cellular RNA was isolated from HTB15 and normal human Astrocyte cells using Trizol (Invitrogen, CA, USA). The RNA quantity was analyzed using the Nano Drop ND1000 (SOP N° TAL009) and RNA quality checked using a Bio-analyzer 2100 (Agilent Technologies, CA, USA).

Sample amplification was performed with 200 ng of total RNA using Agilent Technologies Quick Amp Labeling Kit One Color to generate complementary RNA (cRNA) for oligo microarrays. cRNA microarray analysis was processed using a Whole Human Genome Oligonucleotide Microarray (G4112A, 41,000 genes; Agilent Technologies, CA, USA) according to the manufacturer’s instructions.

#### Microarray hybridization

To prepare samples for microarray analysis, slides were hybridized in buffer containing fluorescencelabeled cRNA at 60°C, 17 h using HS Pro hybridization station. Slides were washed once with 63× SSPE buffer containing 0.005% N-lauryl sarcosine, 1 min at room temperature followed by a 1 min wash using pre heated (37°C) 0.06×SSPE buffer containing 0.005% N-lauryl sarcosine. The final slide wash was performed for 30 sec using acetonitrile.

#### Image and data extraction

Fluorescence signals from hybridized microarrays were detected using an Agilent and DNA microarray scanner with a resolution of 5l M and using Agilent Feature Extraction Software (FES). FES determines feature intensities and normalized ratios by linear LOWESS with background subtraction, rejects outliers and calculates statistical confidences (P-values). Hybridization signals with P value less than 0.001 were considered significant. Only genes differentially expressed in the three repeat experiments were considered as relevant genes.

## Results

### Two-dimensional difference gel electrophoresis (2D-DIGE) and protein identification

**Figure 1:**
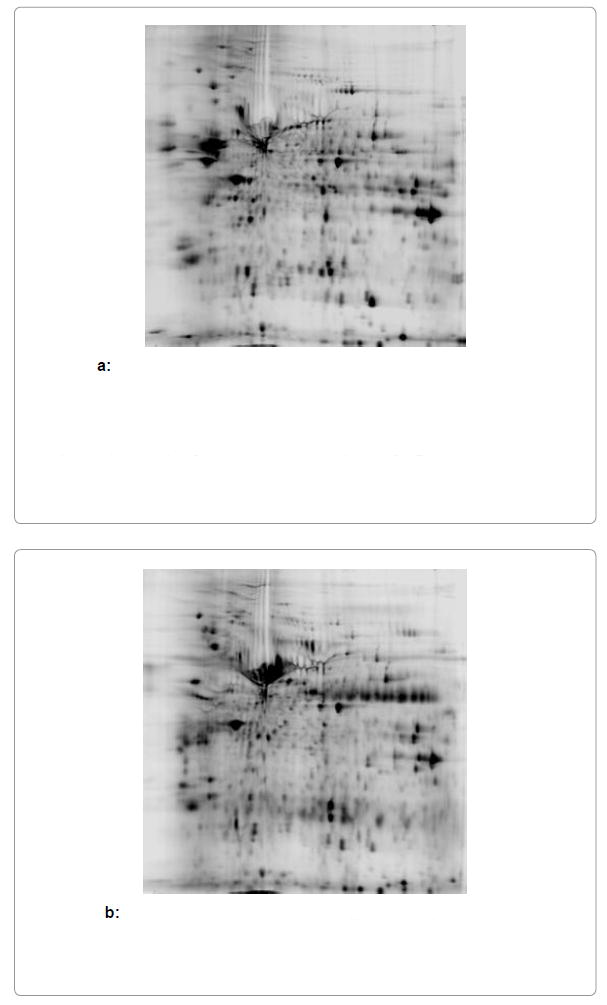
Two-dimensional difference gel electrophoresis image of human normal Astrocyte cell lysate. Lysate was denatured in buffer and solubilized in Tris-HCl, pH 8.8. Gel loading was standardized by total protein (Lowry method). Samples were labeled with cy3/cy5 dye and protein separated using IEF followed by SDS-PAGE. Color image was converted to black and white to analyze volume ratio of cancer/normal samples (n=3). Based on this analysis, candidate protein spots differing notably between GBM and Astrocyte cells were selected for further analysis. Two-dimensional difference gel electrophoresis image of Glioblastoma Multiforme cell lysate. Lysate was processed analogously to methods employed for human normal Astrocyte cell lysate and results interpreted similarly (Figure. 1a).

**Figure 2:**
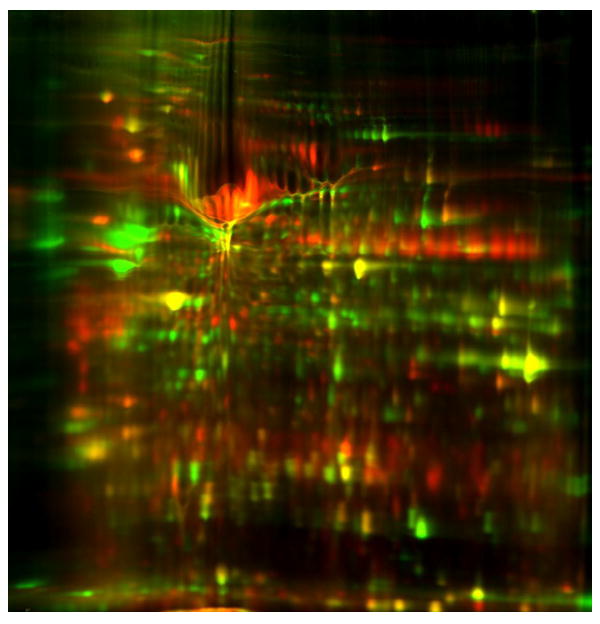
2D-DIGGE Gel picture of the Astrocyte (Green) and Glioblastoma (Red) sample. Original two-dimensional difference gel electrophoresis (2D-DIGE) image in color prior to conversion to black and white depicting protein in lysates from normal Astrocyte cells (green) and Glioblastoma Multiforme cells (red). These lysates were prepared according to methods outlined in Figure 1a.

**Figure 3:**
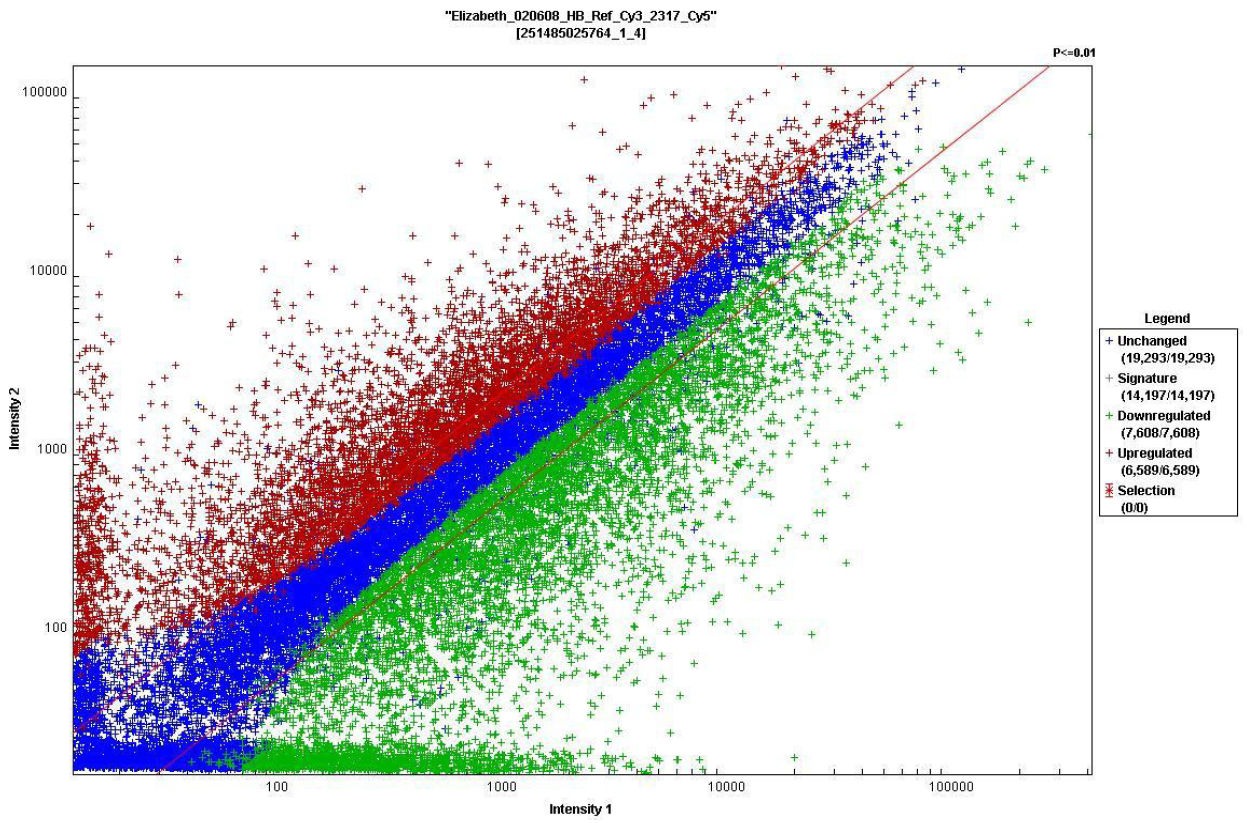
Microarray data indicating genes upregulated and downregulated in Glioblastoma Multiforme cells relative to human normal Astrocyte cells. mRNA was converted to fluorescently labeled cRNA and analyzed using a Whole Human Genome Oligonucleotide Microarray (G4112A, 41,000 genes; Agilent Technologies), according to manufacturer’s instructions.

To identify biomarkers of GBM, we profiled the proteome for differentially expressed proteins in GBM cells versus human normal Astrocyte cells by separating proteins based on pI and molecular weight using 2D-DIGE (Figures 1A and 1B). A large number of protein spots showed notable differential expression between the GBM cells and normal Astrocytes (Figures 1A,1B and 2) but 1 protein spot with most dramatic change was selected for further analysis. The mass spectrometry analysis of this spot revealed the nature of the differentially expressed protein (Table 1). We further investigated the differential expression of the gene coding for this protein by microarray analysis. It is notable that this protein, which was identified as GRP 78, and was largely differentially expressed between GBM and normal Astrocytes, also had its corresponding gene demonstrate higher fold change in GBM cells (Figure 3).

## Discussion

With conventional molecular biological approaches, studies on proteins can only be conducted on a limited number of proteins. Advances in proteomic analysis now enable direct monitoring of global changes in protein expression and post- translational modifications, which will help to identify new biomarkers for GBMs and potentially provide more insight into the treatment of GBMs.

In particular, 2D-DIGE utilizes mass and charge-matched spectrally resolvable fluorescent dyes (Cy3 and Cy5) to label two different protein samples in vitro prior to two-dimensional electrophoresis. To date, 2D/DIGE is still one of the central technologies in proteomics for the separation and differential comparison of thousands of proteins in a complex mixture [21-23].

The protein identified by this novel technique has to be further investigated for its role in GBMs and therapeutic and prognostic use against different therapies. Cancer cells adapt to chronic stress in the tumor microenvironment by inducing the expression of GRP78/BiP, a major endoplasmic reticulum chaperone with Ca2^+^-binding and antiapoptotic properties. GRP78 promotes tumor proliferation, survival, metastasis and resistance to a wide variety of therapies. Thus, GRP78 expression may serve as a biomarker for tumor behavior and treatment response. Combination therapy suppressing GRP78 expression may represent a novel approach toward eradication of residual tumors. Furthermore, the recent discovery of GRP78 on the cell surface of cancer cells, but not in normal tissues, suggests that targeted therapy against cancer via surface GRP78 may be feasible [24,25]. Analysis of more samples for further characterization and genomic microarray analysis will be the next step for studying diagnostic, therapeutic and prognostic approach for this deadly cancer whose suffers have nearly one hundred percent mortality rate.

## Figures and Tables

**Table 1:** Differentially expressed protein GRP78 in GBM sample identified by MALDI mass spectrometry after 2D-DIGE analysis.

Spot Label	Protein Name	Accession Number	Protein MW	Protein PI	Fold of Change
Spot	Glucose regulated protein 78	P11021	78 kDa	5.10	2
